# AMulet 2.0 for Verifying Multiplier Circuits

**DOI:** 10.1007/978-3-030-72013-1_19

**Published:** 2021-02-26

**Authors:** Daniela Kaufmann, Armin Biere

**Affiliations:** 8 Eindhoven University of Technology, Eindhoven, The Netherlands; 9 Aalborg University, Aalborg East, Denmark; Johannes Kepler University, Linz, Austria

## Abstract

AMulet 2.0 is a fully automatic tool for the verification of integer multipliers using computer algebra. Our tool models multiplier circuits given as and-inverter graphs as a set of polynomials and applies preprocessing techniques based on elimination theory of Gröbner bases. Finally it uses a polynomial reduction algorithm to verify the correctness of the given circuit. AMulet 2.0 is a re-factorization and improved re-implementation of our previous multiplier verification tool AMulet 1.0.

## 1 Introduction

Formal verification of arithmetic circuits is important to prevent issues like the famous Pentium FDIV bug [28]. Up to now there have been many attempts to verify these circuits, but even today the problem of fully automatic verification of arithmetic circuits, and especially multipliers, is still considered to be hard.

Methods based on decision diagrams [6] rely on manual structural decomposition of the multiplier. Approaches based on satisfiability checking (SAT) are not scalable [3]. Recently progress has been made using theorem provers [29]. However, the multipliers have to be given as SVL netlists, which relies on preservation of hierarchical information.For flattened gate-level multipliers the currently most successful technique uses algebraic reasoning [7, 15, 17, 25, 26]. In this line of work the circuit is modeled as a set of polynomials and the specification is then checked to be implied by the circuit polynomials. For non-experts Chap. 2 of [15] might serve as introduction to bit-level verification using computer algebra.

In our approach [17] we apply a combination of SAT solving and computer algebra. Certain parts of the multiplier, i.e., complex final stage adders that are generate-and-propagate (GP) adders [27], are hard to verify using computer algebra, but are easy to verify using SAT solvers [21]. Therefore we apply adder substitution [17] and replace complex final stage adders by simple ripple-carry (RC) adders. The equivalence of the adders is verified using SAT solvers. The correctness of the simplified multiplier is shown using computer algebra [17].

This tool paper presents AMULET 2.0, a successor of AMULET 1.0 [17, 19]. AMULET 2.0 reads multipliers given as and-inverter graphs (AIG) [22] and fully automatically applies adder substitution and verifies the (simplified) circuit. Furthermore, certificates can be generated in the Nullstellensatz proof format [16] or in the practical algebraic calculus (PAC) [20] to validate the verification results.

* This work is supported by the LIT AI Lab funded by the State of Upper Austria.

AMulet 2.0 is a modular C++ re-implementation of AMulet 1.0 (while AMulet 1.0 consists of a single C file). AMulet 2.0 is not only a standalone tool but also serves as a polynomial reasoning framework, i.e., parts can easily be integrated into different workflows, cf. Sect. 4. AMulet 2.0 still provides the same functionality as AMulet 1.0, but with improved algorithms, cf. Sect 5, based on the same theory [15,17]. In this paper we focus on novelties of AMulet 2.0 and refer the reader to [19] for an introduction to AMulet 1.0.

## 2 Circuit Verification using Computer Algebra

AMulet 2.0 takes as input signed or unsigned integer multipliers $C$, given as AIGs, with $2n$ input bits $a_0, \ldots, a_{n-1}$, $b_0, \ldots, b_{n-1} \in \{0,1\}$ and output bits $s_0, \ldots, s_{2n-1} \in \{0,1\}$. We denote the internal AIG nodes by $l_1, \ldots, l_k \in \{0,1\}$. Let $\mathbb{Z}[X] = \mathbb{Z}[a_0, \ldots, a_{n-1}, b_0, \ldots, b_{n-1}, l_1, \ldots, l_k, s_0, \ldots, s_{2n-1}]$. The multiplier $C$ is correct iff for all possible inputs $a_i$, $b_i \in \{0,1\}$ the specification $\mathcal{L} = 0$ holds:

$$\mathcal{L} = -\sum_{i=0}^{2n-1} 2^i s_i + \left(\sum_{i=0}^{n-1} 2^i a_i\right)\left(\sum_{i=0}^{n-1} 2^i b_i\right) \quad (1)$$

For signed multipliers the most significant bits $s_{2n-1}$, $a_{n-1}$, and $b_{n-1}$ determine the sign and the weights have to be negated, i.e., $2^{2n-1}$ becomes $-2^{2n-1}$.

The semantics of each AIG node implies a polynomial relation, e.g., $u = v \land \neg w$ implies $-u + v - vw = 0$. Let $G(C) \subseteq \mathbb{Z}[X]$ be the set of polynomials that contains for each AIG node the corresponding polynomial relation. Additionally, all variables $x \in X$ are Boolean and we enforce this property by the set of *Boolean value constraints* $B(X) = \{x(1-x) \mid x \in X\} \subseteq \mathbb{Z}[X]$. The polynomials in $G(C) \cup B(X)$ are ordered according to a lexicographic order, such that the output variable of a gate is always greater than the inputs of the gate [23].

Let $J(C) = \langle G(C) \cup B(X) \rangle \subseteq \mathbb{Z}[X]$ be the ideal generated by $G(C) \cup B(X)$. The circuit fulfills its specification if and only if we can derive that $\mathcal{L} \in J(C)$ [17]. We showed in [17] that $G(C) \cup B(X)$ is a D-Gröbner basis [2] for $J(C) \subseteq \mathbb{Z}[X]$. Thus, the correctness of the circuit can be established by reducing $\mathcal{L}$ by the polynomials $G(C) \cup B(X)$ and checking whether the result is zero.

However, simply reducing the specification by $G(C) \cup B(X)$ leads to large intermediate results [24]. Hence, we eliminate variables in $G(C) \cup B(X)$ prior to reduction to yield a more compact D-Gröbner basis [17], which boils down to simple substitutions, but relies on the elimination theorem of Gröbner bases [9].

## 3 Usage

AMulet 2.0 is available at http://fmv.jku.at/amulet2 and is published as open source under the MIT license. AMulet 2.0 relies on the AIGER library [5] and the GMP library [10]. The AIGER library is provided together with the source code of AMulet 2.0, the GMP library needs to be pre-installed by the user. AMulet 2.0 is compiled executing "`./configure.sh && make`".

In a complete workflow one should first apply adder substitution, using the *substitution mode* of AMulet 2.0, to make sure that a potential complex final stage adder is replaced by a simple RC adder. Afterwards, one of the two modes, the *verification mode* or *certification mode*, can be applied to verify the (simplified) multiplier, which we will call in the following *rewritten* multiplier. If it is known that the final stage adder is not a complex GP adder, the substitution step can be omitted. We present a complete demonstration for the unsigned 64-bit multiplier `<bpwtcl.aig>`, which is included in the complementary material [14]. The output of AMulet 2.0 can be seen in the corresponding log-files that are also included in the artifact.

**Adder Substitution.** First we apply adder substitution by running

```
./amulet -substitute bpwtcl.aig miter.cnf rewritten.aig [options]
```

If the multiplier computes multiplication of signed integers the option "`-signed`" has to be involved, because the signedness is part of the circuit specification.

If adder substitution can be applied successfully, the generated miter is written to `<miter.cnf>` and the rewritten multiplier to `<rewritten.aig>`. Otherwise, the input multiplier will be written to `<rewritten.aig>` and a trivially unsatisfiable CNF is written to `<miter.cnf>`. The file `<miter.cnf>` has to be given to a SAT solver, e.g. Kissat [4], which is then expected to return *unsatisfiable*. The rewritten multiplier can be verified or certified using AMulet 2.0.

**Verification.** Verification is executed by

```
./amulet -verify rewritten.aig [options]
```

As for adder substitution, one has to invoke the option "`-signed`" for signed multipliers. Furthermore, the option "`-no-counter-examples`" is available, which turns off generation and saving of counter examples in `<rewritten.cex>`, in the case when the multiplier in `<rewritten.aig>` is incorrect.

**Certification.** Certification is applied using

```
./amulet -certify rewritten.aig out.pol out.prf out.spc [options]
```

In this mode, AMulet 2.0 verifies the multiplier and automatically generates proof certificates, which can be checked by corresponding proof checkers. AMulet 2.0 supports two proof formats, Nullstellensatz proofs [1,16] and PAC proofs [20] based on the polynomial calculus [8]. The default proof format is the Nullstellensatz proof, because it generates smaller proof files and is faster to check. Proofs in the PAC format can be generated using the option "`-pac`". All options of the verification mode are available too.

The proofs are stored in the provided files `<out.pol>`, `<out.prf>`, and `<out.spc>`. The file `<out.pol>` contains the gate constraints, the second file `<out.prf>` the core proof in the selected proof format and the third file `<out.spc>` the specification of the multiplier. The generated proofs can be given to the proof checkers Nuss-Checker [16] for Nullstellensatz proofs or to the proof checkers Pacheck [20], or Pastèque [20] for PAC proofs.

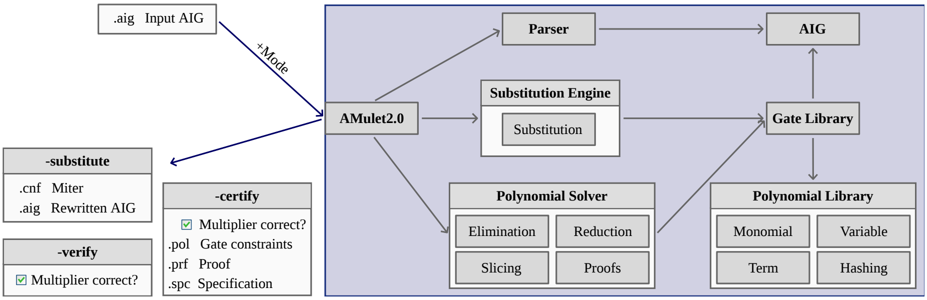

**Fig. 1.** Architecture of AMULET 2.0.

## 4 AMulet 2.0

In this section we present the architecture of AMULET 2.0 and discuss novel optimizations. The design of AMULET 2.0 is shown in Fig. 1. In contrast to AMULET 1.0, which consists of one single C file, AMULET 2.0 is split into components, which also allows to integrate only parts, e.g., the polynomial library or the polynomial solver, in different workflows, cf. the provided demos in the artifact [14]. AMULET 2.0 is implemented in C++11 and consists of around 6 000 lines of code. It relies on the AIGER library [5] to process the given AIG and the GMP library [10] to represent large integers.

The mode of AMULET 2.0 is triggered by the command line input, cf. Sect. 3. In substitution mode, AMULET 2.0 parses the AIG, allocates the internal gate structure, and invokes the substitution engine for adder substitution. In verification mode, AMULET 2.0 reads the AIG and initializes the gate structure. Afterwards, the circuit is verified in the polynomial solver using polynomial operations of the polynomial library. In certification mode proofs are generated in addition. In the following we present the individual components of AMULET 2.0.

**Parser Module** AMULET 2.0 checks whether the given AIG circuit fulfills the requirements described in Sect. 2, i.e., the AIG circuit has an even number of inputs and an equal number of outputs. The AIG module wraps functions of the external AIGER library that are needed to process the input file.

**Gate Library** After parsing we allocate a gate for each AIG node, which includes structural information, such as dependencies, or whether the gate represents an input/output or an XOR-gate. Furthermore, each gate is linked to a unique variable. If the given AIG is verified or certified, AMULET 2.0 also initializes the gate constraints and creates the specification polynomial $\mathcal{L} \in \mathbb{Z}[X]$.

**Substitution Engine** In substitution mode, AMULET 2.0 applies heuristic pattern matching to identify GP adders [17]. In AMULET 2.0 we enhanced the identification heuristics and cover special cases that are not considered in AMULET 1.0. Thus, AMULET 2.0 is able to detect more GP adders than AMULET 1.0. After a positive GP pattern match, AMULET 2.0 generates an equivalent RC adder and replaces the GP adder by the RC adder. A bit-level miter is generated in CNF to verify the equivalence of the adders. The rewritten multiplier and the CNF miter are printed to the provided output files.

**Polynomial Solver** The polynomial solver is based on the solving engine of AMulet 1.0 [19] and is used to verify or certify the given multiplier. In a nutshell, the polynomial solver first applies preprocessing by eliminating selected variables. Afterwards, the remaining variables are ordered into column-wise slices, such that we can apply our incremental verification algorithm [18], where we split the specification $\mathcal{L}$ into multiple polynomials and verify the multiplier by deriving the correctness of each slice using polynomial reduction. The necessary polynomial operations are implemented in the **Polynomial Library**.

In AMulet 2.0 we eliminate variables before ordering them, while in AMulet 1.0 it is the other way around. We eliminate all internal gates of the XOR-structures and all single-parent nodes in the AIG. Thus, fewer variables are considered for ordering, which improves computation time of AMulet 2.0.

Furthermore, we include a novel XOR-based slicing approach in AMulet 2.0, which relies on the fact that many multiplier architectures use XOR-skeletons to compute the output bits. We identify these skeletons and assign all nodes of a skeleton to the same slice. Gates occurring between XOR-skeletons are assigned to the smaller (less significant) slice. Hence, after two iterations all slices are fixed, which improves slicing compared to AMulet 1.0. All variables that are not assigned to slices, e.g., gates used to compute the partial products in Booth encoding [27], are eliminated from the gate structure.

In few cases, where we cannot identify XOR-skeletons, e.g., in multipliers containing a carry-select adder, we fall back on the slicing approach of AMulet 1.0: We slice based on input cones and eagerly move gates between slices to reduce the number of carries, by iterating multiple times over the variables.

After assigning gates to slices, AMulet 2.0 reduces the slice-wise specifications incrementally by the sliced gate constraints and checks whether the final result is zero, following the implementation of AMulet 1.0. If the final remainder is not zero, AMulet 2.0 detects counter examples, i.e., input assignments for which the multiplier circuit computes an incorrect result.

In certification mode, AMulet 2.0 tracks polynomial operations in the selected proof format, i.e., Nullstellensatz or PAC, and prints gate constraints, the generated proof, and the specification $\mathcal{L}$ to the provided files.

**Polynomial Library** The polynomial library implements the arithmetic operations for addition and multiplication of polynomials (by constants), and division by terms. Since all variables represent Boolean values, we always reduce exponents greater than one automatically to one, i.e., we assume $x \cdot x = x$.

Polynomials are represented as linked lists of monomials. Each monomial consists of a coefficient, represented using the GMP library, and a term. Terms are linked lists of variables, which are internally shared using a hash table.

In AMulet 1.0 we do not share monomials and make hard copies in the few occasions when a monomial needs to be copied. This has the benefit that we can simply modify coefficients of the monomials, e.g., during addition. In our experiments we observed that allocating new GMP objects is actually quite time consuming, and therefore we now share monomials in AMulet 2.0, using reference counting, which decreases verification time by a factor of two.

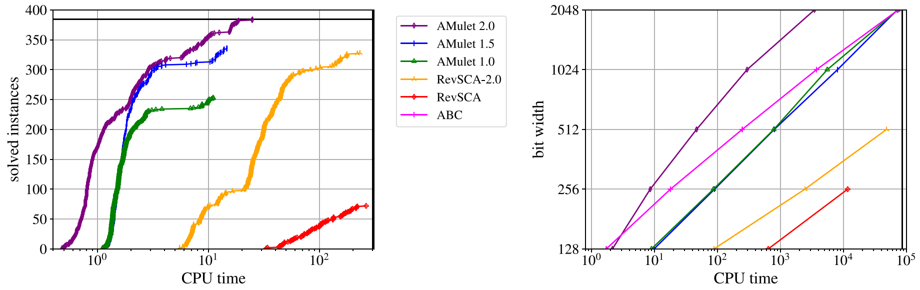

**Fig. 2.** Verification of AOKI multipliers (left) and of large multipliers (right), in seconds.

## 5 Evaluation

In our experiments we use an Intel Xeon E5-2620 v4 CPU at 2.10 GHz (with turbo-mode disabled) with a memory limit of 128 GB. The time is listed in seconds (wall-clock time). We compare AMulet 2.0 to our previous tool AMulet 1.0 and to the most recent related work RevSCA, RevSCA-2.0 [25] and ABC-based work of [7] on multiplier verification using computer algebra, where circuits are given as AIGs. The tool of [26] is not yet available. We consider two versions of AMulet 1.0: (i) AMulet 1.0 as published in [17], (ii) AMulet 1.5 a slightly improved version [13] with new heuristics for detecting GP adders. The experimental data is included in the artifact [14].

In our first experiment we consider the comprehensive AOKI benchmark set [12], which provides 384 signed and unsigned integer multiplier architectures up to input bit-width 64, also covering Booth encoding. We consider all 384 architectures of bit-width 64. The time limit is set to 300 seconds. The results are shown on the left side of Fig. 2, where it can be seen that AMulet 2.0 is the only tool that is able to verify the complete benchmark set. RevSCA only supports verification of unsigned integers. ABC-based work of [7] uses an optimization, which only works for simple multiplier architectures. Enabling this optimization on the more involved AOKI benchmarks leads to incompleteness. Without enabling it [7] either produces a segmentation fault or exceeds the time limit. Thus there are no results for [7] on the left side of Fig. 2.

In our second experiment we generate benchmarks of simple multipliers up to input size 2 048, using scripts by Arist Kojevnikov [11]. The time limit is set to 86 400 seconds (24 h) and the results are shown on the right side of Fig. 2. It can be seen that AMulet 2.0 outperforms all competitor tools and is an order of magnitude faster on large multiplier circuits.

## 6 Conclusion

We presented AMulet 2.0, a fully automatic tool for verifying multiplier circuits given as AIGs. AMulet 2.0 is a re-factorization and re-implementation of our previous verification tool AMulet 1.0 [17,19] and successfully verifies a large set of multiplier architectures. In the future we want to directly integrate a SAT solver into AMulet 2.0 and provide language bindings, e.g. for Python.
